# Exploring barriers and solutions in advancing cross-centre population data science

**DOI:** 10.23889/ijpds.v4i1.1109

**Published:** 2019-08-05

**Authors:** KH Jones, SM Heys, H Daniels, DV Ford

**Affiliations:** 1 Population Data Science, Swansea University Medical School, Singleton Park, Swansea SA2 8PP

## Abstract

**Introduction:**

It is widely acknowledged that population health and administrative data, especially when linked at the individual level, hold great value for research. Cross-centre working between data centres providing access to such data has the potential to further increase this value by effectively expanding the data available for research. However, there is limited published information on how to address the challenges and achieve success. The aim of this paper is to explore perceived barriers and solutions to inform developments in cross-centre working across data centres.

**Methods:**

We carried out a narrative literature review on data sharing and cross centre working. We used a mixed methods approach to assess the opinions of members of the public on cross-centre data sharing, and the views and experiences of among data centre staff connected with the UK Farr Institute for Health Informatics Research.

**Results:**

The literature review uncovered a myriad of practical and cultural issues. Our engagement with a public group suggested that cross-centre working involving anonymised data being moved between established centres is considered acceptable. The main themes emerging from discussions with data centre staff were dedicated resourcing, practical issues, information governance and culture.

**Conclusion:**

In seeking to advance cross-centre working between data centres, we conclude that there is a need for dedicated resourcing, indicators to recognise data reuse, collaboration to solve common issues, and balancing necessary barrier removal with incentivisation. This will require on-going commitment, engagement and an academic culture change.

## Introduction

Population data science [1] is the multi-disciplinary field concerned with enabling and using large-scale datasets of health and wider administrative data for research. Examples of health data include routine primary and secondary care records, population surveys, condition-specific cohorts; wider administrative data include education, housing and social service records, amongst others. These datasets have proven value in research, especially in linked form, as evidenced by even just a cursory glance at the many publications listed by organisations specializing in providing access to such data in accordance with jurisdictional regulatory and governance requirements [2-4]. We refer to such organisations as data centres, and we use the term data-intensive research to refer to studies using large-scale datasets separately or in linked form for population data science. Data are often made accessible in anonymised form, a definition dependent on jurisdictional legislation. Despite many successes, there are limits to what can be achieved using data collated via individual centres, or within separate jurisdictions. This can be the case with rare conditions, extremes of age, infrequent prescriptions and other factors that may limit sample size. Furthermore, being able to use data from across settings can enable comparisons, such as with other geographical areas and healthcare systems, and provide efficiencies of scale. As a result, there is a push for cross-centre working in data-intensive research.

Data are being shared more widely than ever before and whilst there are risks, the benefits of exchanging data in a controlled way are potentially huge. The drive for data sharing can be seen from a consideration of major funding body policies. Two Research Council examples from the UK are given here to demonstrate the point. The Medical Research Council (MRC) data sharing policy [5] sets out that the *‘overarching policy aim for data sharing is to maximise the life time value of research data assets for human health and to do so in a way that is timely, responsible with as few restrictions as possible, and consistent with the law, regulations and recognised good practice.’* The policy puts forward that researchers must make clear provision for data sharing when planning and executing research. Outlined in the document is that data should be shared in a timely and responsible manner and that data sharers should receive full and appropriate recognition by funders, their academic institutions and new users for promoting secondary research. Similarly, the Economic and Social Research Council (ESRC) policy [6] states that research data are a *‘public good, produced in the public interest which shall be made openly available and accessible with as few restrictions as possible.’* Furthermore, it states that non-deposit of research data should be an exception and that grant holders must address the issues that could limit data sharing opportunities from the very start of the project.

In relation to cross-centre working between data centres more specifically, the Australian Population Health Research Network (PHRN) is a national data linkage network, supporting state and territorial data centres, and enabling data from across the nation to be used for research [4]. The Canadian government has recently invested in the development of a National Data Platform [7] and the UK has seen the establishment of Health Data Research UK [8]. All these initiatives place great emphasis on the importance of cross-centre working to advance research. However, there are challenges to be overcome if cross-centre working is to become a norm in this sphere of work. In the context of this paper, we refer mainly to cross-centre working, rather than data sharing, because not all models of working between centres involve data movement and pooling, but may rely on federated data access.

### Aim and scope of this paper

The aim of this paper is to explore perceived barriers and solutions in cross-centre working in population data science. The work is based on the Advancing Cross-centre Research Networks study (referred to as ACoRN) which was commissioned by the UK Farr Institute of Health Informatics Research [9] and conducted between January and December 2017. As such, the engagement with data centre staff is based on the Farr Institute (of which there were four centres: one in each of Wales and Scotland and two in England) but the findings will be discussed in the wider context for generalisability. ACoRN relates to the sharing of anonymised, individual-level data, which within the UK, is outside data protection legislation [10-12]. The GDPR definition of anonymised is given in Recital 26: ‘information which does not relate to an identified or identifiable natural person or to personal data rendered anonymous in such a manner that the data subject is not or no longer identifiable’ [10].

## Methods

### Literature review

A narrative literature review was conducted by SH at the start of the study period. This was based on structured search terms making use of major search engines such as Google Scholar, Web of Science, Scopus and PubMed, as well as government and regulatory body websites. The search terms used were ‘health and cross centre working’, ‘cross centre research’, ‘barriers to cross centre working’, ‘incentives to cross centre working’ ‘communities of practice’ and ‘collaborative working’. A total of 46 articles were identified within the search parameters and, of this number, 12 were excluded on the basis of not being relevant to the project. The content of the publications was analysed manually by two researchers (SH and HD) to draw out common themes. The information gained was used to shape engagement with the public and data centre staff.

### Public opinions on cross-centre data sharing

While there is much work with the public on the social acceptability of data-intensive research, it is our view that cross centre working is more specific and warrants further engagement activity. Opinions on cross-centre working were gained through face-to-face discussion with the Swansea University-based Consumer Panel for data linkage on 25th January 2017 [13]. This is an active group of 18 members of the public who advise on the use of population data for the SAIL databank and associated initiatives [14]. At the start of the session the panel were given a short, written, fictional scenario involving pooling anonymised data from two centres: one in each of England and Wales, and asked to consider the acceptability of this kind of cross-centre working. The discussion was led by SH; notes were taken by HD and the information was analysed manually by SH and HD.

### Views and experiences of cross-centre working among data centre staff

Data centre staff work on a day-to-day basis either carrying out research or enabling it to happen. As such we consider it essential to factor in their experiences for learning and to ensure inclusivity for buy-in on cross-centre working. We engaged with staff in a variety of ways to build up the information. A stakeholder workshop was held at the Informatics for Health conference [15] in April 2017 with an international group (N=12), comprised of researchers, analysts, managers and governance specialists from countries including Italy, Canada, England and Ireland. The workshop was facilitated by KHJ, SH and HD. Participants took part in an activity to suggest barriers and solutions in cross-centre working. These written suggestions were subjected to manual thematic analysis and grouped by consensus of three researchers (KHJ, SH & HD). They were used to create an anonymous survey conducted (by SH) to ask Farr staff to rank the barriers and solutions in cross-centre working identified in the stakeholder workshop (N=99, of which 23 responded). Further consultations with Farr Institute staff (N=20) were conducted between May and September 2017 to gain individual views in more depth. These were led by SH and took the form of one-to-one, unstructured interviews with an open discussion to explore each interviewee’s opinions and experiences of cross centre working. The discussions were noted and themed by SH and HD.

## Results

### Findings from the literature on data sharing

Since cross-centre working between data centres is still novel and developmental, most of the literature we found relates to data sharing in general, but still yields valid points for consideration. Practical issues have long been highlighted as barriers to data sharing, and many of these are even more relevant to cross-centre working because of the need for data compatibility for combined analyses. These include actively facilitating data sharing, the need for dedicated funding and incentivising data custodians to share information as well as providing the technical infrastructure to allow sharing to occur [16]. A systematic review in public health uncovered many practical barriers to data sharing, including: data not retained; coding differences; restrictive data formats; absence of metadata and standards; lack of suitable technical infrastructure; no incentives; lack of resources; opportunity costs; disagreement on data uses; absence of guidelines on ownership and copyright; restrictive policies; protection of privacy; and a lack of trust and reciprocity [17]. Furthermore, it is recognised that many barriers can only be overcome by dialogue aimed at generating consensus on policies and instruments for data sharing [17]. The need for practical solutions such as a search portal across repositories, consistent standards, policies and review processes, and the ability to track dataset reuse were proposed over a decade ago and remain largely unaddressed [18].

Data sharing has been described as a *‘complex messy and non-linear process’* and a *‘jigsaw of conflict and cooperation’*, such that protocols and procedures alone are unlikely to mitigate the risks [19]. Academic institutional factors are seen as hindering data sharing including the culture relating to publication as a yardstick of success. Researchers are incentivised to publish papers and not to share data actively and furthermore there are a number of disincentives to sharing, for example, others using the data to get ahead [20]. It has been proposed that if publishing the data were held to be the measure of success rather than publishing academic articles themselves the picture may change [18]. Monitoring the levels of data exchange could be an active incentive to allow researchers to quantify the value of their contribution and motivate future sharing [16]. The literature chronicles the technical, organisational, institutional and political obstacles to data sharing, but a core message is that technical cleverness and rule-driven agreements will never be enough to overcome cultural obstacles. It is proposed that cross-agency communities of practice are required to confront practices protective of pre-existing ways of working and resource allocations. This in itself needs effort to maintain direction since resistance and subversion may occur if practices are changed which result in bureaucratic frustration and compromise rather than creating positive change for analysts and researchers. There is an identified need for a framework that builds familiarity, trust, mutual purpose and dependency across a data sharing community of practice that allows risks to be understood and mitigated [19]. Whilst technical challenges are not trivial and solutions are essential, a key message in the literature is that cultural factors are likely to be harder to solve [18, 21].

### Findings from the literature on cross-centre working

We go on to consider literature specifically on cross-centre working in population data science. The International Journal of Population Data Science (IJPDS), has published a special issue call for manuscripts on the topic of cross-centre working, including outcome-based research using data from more than one data centre, and methodological developments to overcome the hurdles and enable data to be used more effectively across centres [22]. We draw from this to gain information on overcoming barriers developing and solutions.

A UK study took the form of a case study in distributed team science in research using routine health records. The research team sought to carry out the study using data from centres in Wales and Scotland and they explored the pros and cons of five possible access models. These were one pooling and four federated options: i) movement of data to one of the centres; ii) data remaining at each centre and accessed by a single analyst; iii) data remaining at each centre and accessed by separate analysts employed at each centre; iv) data access by an analyst using a brokered remote connection between the centres; and v) single analyst directing queries across distributed data. For data governance and resource reasons, they settled on a combination of models ii) and iii) whereby analysts employed at each centre were given simultaneous access to data at both sites using the same algorithm [23].

Another UK-based study discussed the challenges in accessing routinely collected data from multiple providers in England and Wales in the following categories: the data application process; project timelines; dependencies and considerations related to consent; Information Governance; and contractual issues. Among these there were differing data provider requirements, and lengthy, changeable approval processes. There were Information Governance challenge to reach and provide the evidence of reaching the required standard. This was further complicated because multiple data providers were involved and no single way to demonstrate good practice. The authors recommended more effective communications in the form of: a continuing dialogue between the research community and data providers; similarly, between data providers and funders; and the constructive sharing of experiences by users of routine data [24].

Challenges associated with cross-jurisdictional analyses using administrative and health data were examined by a team of researchers in Canada. The limited capacity to share and use data across jurisdictional boundaries, was attributed in part to cumbersome approval and access procedures, and to the lack of harmonisation in data sharing laws across provinces and territories, resulting in risk averse interpretations. Furthermore, inconsistencies among variables and indicators make it difficult to compare research findings among jurisdictions. The authors compared and contrasted data access procedures in three Canadian jurisdictions, and described how they addressed the challenges. They saw important solutions in cross-centre data working being to build capacity for among analytic staff with ground-level familiarity with available data, fostering expertise in adapting code and variable definitions, and developing strong communication skills for working across jurisdictional borders to work around the barriers [25].

Further articles accorded with the points raised and some stressed additional issues. In describing a federated model for chronic disease surveillance, authors highlighted challenges in data heterogeneity across jurisdictions and changes in data quality over time threatening the production of standardized disease estimates. They proposed the need to balance comprehensive reporting with differing jurisdictional disclosure requirements to protect privacy [26]. A cardiac registry collects detailed data prospectively from the point of care, rather than relying on data feeds. It circumvents some common data format problems by having pre-defined fields and common definitions. However, these sorts of issues resurface to some extent when linking the registry to routine data [27]. Working on cancer care, authors proposed that it takes a village to understand inter-sectoral care using administrative data across jurisdictions. They advised that sectoral participation could be maximised by ensuring decisions were properly inclusive of all participating jurisdictions, by the use of living documents to track the decisions, and by careful recording of data quality and availability differences [28]. In a multi-centre epidemiological study, authors identified the need for agreed standards, detailed coordination and extensive cooperation with all involved [29], which again stresses the crucial importance of human and cultural issues in making cross-centre working a success.

### Public opinions

Members of the Consumer Panel discussed and provided their views on a fictional cross-centre research scenario relating to dementia. The data of interest included GP information relating to dementia symptoms and medication history, hospital out-patient and in-patient episodes, and genetic markers predisposing to this illness. This hypothetical study would also assess whether geographical and environmental factors were affecting disease rates between the populations of Wales and a region in the north of England. As such, this described a data pooling model involving the exchange of anonymised information between centres rather than the data being held in one centre alone. This scenario was chosen because the Panel members are more familiar with repository models, and we assumed that the pooling of data could be seen as more contentious than federated access. Their viewpoints are summarised under the following questions which were posed to guide the discussion.

i) Do you have any issues with the two sets of data being pooled and the information being accessible across the two institutions?

The panel raised the issue of differing data formats and consistency across the centres and whether this would be a barrier to the exchange of information. Their view was that this is a significant issue for data sharing and needs to be addressed, along with differing access rules across different centres and the challenges that this presents. There was further discussion about information governance approvals and how these need to be compatible across centres and jurisdictions. The consensus view was that as long as the data are effectively anonymised, cross-centre data sharing was generally acceptable.

ii) Do you consider there are any risks to pooling of data in this manner and, if so, whether the benefits outweigh the risks?

The panel raised the need for clear safeguards to be in place for data pooling. They noted that the advantages in combining data from multiple locations are most pronounced in rare conditions where the sample would be otherwise small. They also highlighted greater opportunities to work in line with NHS priorities such as cancer care, dementia and obesity rates and that these should be utilised to justify data sharing where necessary. In terms of risks, a point was raised about which parties could access the data as members would not be content with a commercial company, such as Google, profiting from cross-centre data sharing. Their view was that this would be detrimental to research and would mean that the public would be less willing to share their data.

iii) Should there be any limit to this sort of cross-centre working given that the anonymised information we are considering is not classed as personal data?

The panel’s considered view was that if there is assurance that data are being used responsibly and for a valid reason then there should not be a limit on non-commercial usage provided that the data are anonymised. They noted the need for joint systems and working and appropriate checks and balances to ensure safeguards are in place, and provided this is so, the panel felt assured that further limitations should not apply.

### Views and experiences of data centre staff

We worked with an international group of researchers, analysts, managers and governance specialists to explore views and experiences of cross-centre working. Because the attendees came from a variety of backgrounds, they were asked to consider the opportunities and challenges in cross-centre working in principle, rather than specifically by data sharing model or jurisdiction. This resulted in the identification of a range of barriers and solutions for cross-centre working. There was a perceived lack of information on cross-centre working in general, and of knowing people with similar research interests. From the data management perspective, resource issues ranked highly in terms of time for data preparation along with problems due to differing data formats.

**Figure 1: Ranked barriers to cross-centre working:**
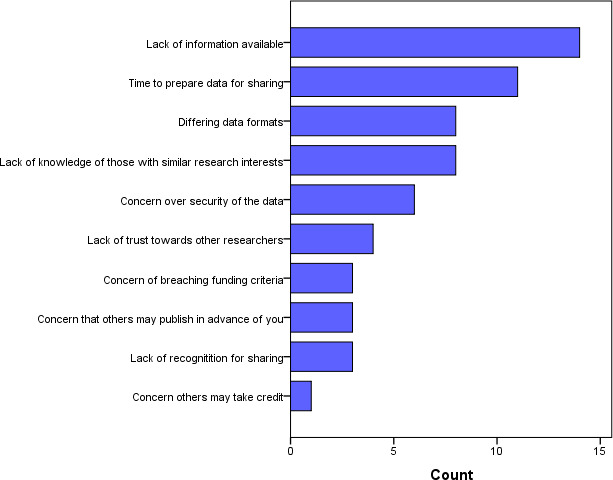
This shows the views of staff (N=23) from Farr Institute centres on barriers to cross-centre working.

**Figure 2: Ranked solutions that would most encourage cross-centre working:**
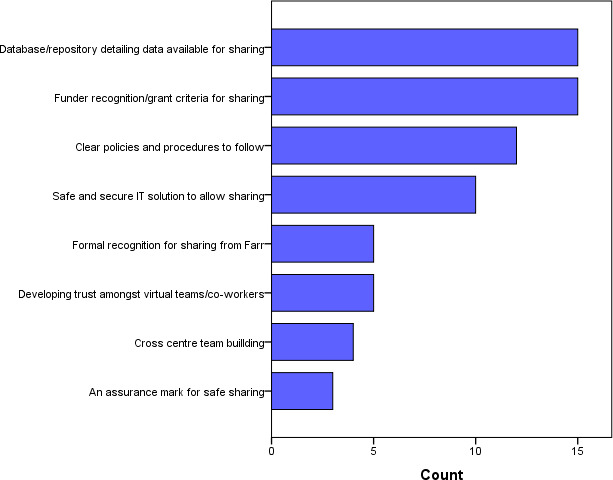
This shows the views of staff (N=23) from Farr Institute centres on solutions for cross-centre working.

The raw lists were grouped into themes by (SH and HD) and incorporated into a short survey across Farr Institute centres to ask staff to rank their top three barriers to cross-centre working, and top three solutions that would most encourage them to work across data centres (Figures 1 and 2).

The lack of information in general featured most highly with 14 of the 23 survey respondents choosing this as the most significant barrier to cross-centre working. This was followed by the time taken to prepare data for sharing (N=11), and then differing data formats and a lack of knowledge of others with similar research interests (N=8 each). Interestingly, topics that could be seen as academic preciousness were ranked among the least important: concern that others may publish in advance (N=3); lack of recognition (N=3); and lowest of all, concern others may take credit (N=1).

In relation to solutions for cross-centre working, two factors were jointly most popular among the survey respondents (N=23). These were the database / repository detailing the data available for sharing (N=15), and funder recognition / grant criteria for sharing (N=15). An assurance mark for sharing was chosen by the fewest people as a potential mode of encouragement (N=3). Among the top four solutions, three can be seen as suggestions to overcome barriers and one on incentivisation. The survey responses might not be representative of all staff at the Farr centres, or more widely, and since they were anonymous we could drill down any further. However, the findings could inform further work with staff in other data centres.

More in-depth views were gained by interviewing staff from Farr centres to discuss issues of their own choosing in cross-centre working. These are summarised by main emerging theme below. Since the survey was anonymous, the degree of overlap between the survey and interview respondents is unknown.

#### Dedicated resourcing

One of the issues highlighted in the interviews was the need for dedicated funding for cross-centre working if it is to be a success. The Farr centres were separately financed with separate accountability to funders. This was viewed as a barrier to sharing as it created a tension between the centres and set them up against each other when bidding for funding and grants. It also meant there was no dedicated resource to cover the expense of cross-centre working. One of the interviewees noted that: *‘There is a real tension between sharing being good in the general sense and sharing being bad in the individual sense. If I have laboriously collected and curated a dataset then it’s hardly surprising that I’m not keen to share it with all and sundry, but I will if the funder makes it a condition to do so.’*

#### Sampling Strategy

Interviewees noted the existence of different operating models in the UK localities and jurisdictions, since the Farr centres did not use a common model. This lack of common infrastructure, and differing formats in their respective datasets, were seen to present technical and compatibility issues for cross-centre working. Further difficulties on this topic included fragmentation among health care data providers and the question of how to create sufficient common ground to move forward. Interviewees said: *‘If we are going to share data, we need common measures of quality to enable us to assess the risks.’* [There is a need for a] *‘clear promise of greater scientific and policy insights arising from analysis of pooled datasets.’*

### Information Governance

Barriers were perceived in the absence of consistency in Information Governance policies between data centres, and in the lack of common external accreditation for data centre operating systems. This was seen to raise difficulties for data controllers in knowing whether they could trust the environments into which they might be placing their data. In terms of review processes for proposed data uses, it was identified that blockages often occur around the interpretation of rules as organisations have differing views on matters of policy. This is further complicated by applications needing to be made to multiple information governance review panels. An interviewee said: *‘A significant barrier to data sharing is a lack of consensus about IG approval.’*

### Culture

The culture within academia in general was seen as a barrier to cross-centre working. This is because targets and achievements are monitored per organisation, with no particular positive indicators for collaborative working. There is also the political element between data centres, in that each centre wants to raise its profile and attract new grant monies. It was felt, that when there is a desire to engage in cross-centre working there is a lack of guidance and assistance to enable this to happen, leaving it seen as too much effort without clear benefits. Trust was seen to be at the crux of the issue when considering whether sharing will be successful. An interviewee observed that: *‘A central register [of datasets] would be very helpful, but per se it won’t encourage sharing and cooperative work because that is more cultural than simply listing what data is available.’*

## Discussion

This novel study has provided new insights by exploring barriers and solutions for cross-centre working in population data science, particularly between data centres specialising in providing access to large-scale, individual-level (and often linked) datasets for research. It was encouraging to note that the members of the Consumer Panel were generally positive about cross-centre working provided that the data were anonymised and safeguarded. Members recognised the benefits of larger data samples for the study of rare conditions, but also highlighted the risks in who might be allowed access to the data, and their discontent with any possibility of data being shared with large data-hungry companies like Google. Although there is a wealth of work with the public on data sharing in general, further public engagement is called for to gain the views of a larger group of people on various models of cross-centre working. The need for dedicated resourcing, practical solutions for data format inconsistencies, streamlining approval processes, and changes to the prevailing academic culture were the main final themes emerging from engagement with data centre staff. It will be crucial to factor staff views into cross-centre developments, since they include the academics and researchers who will initiate studies, and the analysts and data managers who will curate the data on their behalf.

As we noted in the introduction, data sharing and cross-centre working are high priorities, as seen in the remits of large-scale population data science investments and funding body policies. Although the policies generally refer to data sharing, rather than cross-centre working specifically, we argue that it makes sense to consider the incorporation of the data into an established data centre to maximise the opportunities for data reuse through data linkage. Since data reuse is clearly a funder imperative, the question arises as to whether the costs of enabling reuse are properly considered. Our study indicates that often this is not adequately resourced. We propose that if this is not systematically addressed, cross-centre working (as well as data sharing more generally) will not become the norm. To address this issue, there is a need for greater recognition of the work entailed, which could be achieved by funders drawing further upon the knowledge present within data centres to gauge the requirements of data curation for cross-centre working.

The literature shows that there is a myriad of practical issues that must be addressed to facilitate data sharing. As we have seen from our study, many of these are at least equally pertinent to cross-centre working, and they are issues being commonly experienced. Whilst collaboration in overcoming practical barriers is taking place, we propose that is would be valuable to have a dedicated international forum for sharing lessons learned in developing solutions. However, although many issues can be solved by practical means, others can only be fully addressed by dialogue and inclusivity within and across the particular scenarios under consideration. Among the latter would be the specifics of information governance processes that depend on: particular legislative and data governance backdrops where the work is cross-jurisdictional; data provider confidence in the controls on their data once shared to other centres; due diligence processes that vary with organisational policies; and public engagement in relation to differing sharing options, taking into account local culture and political contexts.

The prevailing culture in academia featured strongly in the literature and in our study. We therefore concur that whilst other factors can be challenging, cultural issues are the most difficult to overcome. To begin with, it is often not easy to drill down in order to understand individual motivations or organisational patterns of behaviour. An examination of this topic is far beyond the scope of this paper, other than to say we assume that it is not wholly specific to cross-centre working, but is about human interaction in general. As such, we propose that addressing cultural issues will require a concerted effort including a combination of good communications, inclusive and transparent decision-making, recognition, incentivisation, the development of a shared vision and a clear sense of purpose in cross-centre working. This is a multi-faceted and complex issue, but we suggest two practical steps to work towards its address. Academic institutions and funding bodies need to place quantifiable value on the reuse of data; this could be tracked by means of a persisted data asset number as we have suggested previously in relation to incentivising administrative data reuse [30]. Cross-centre groups need collaboration agreements to acknowledge respective contributions to articles and funding bids, for all contributing staff, not neglecting the work of those who put in effort curating data to enable research.

As noted at the outset, the ACoRN study focused on the sharing of anonymised, individual-level data, and within the UK, this is outside data protection legislation [10-12]. Because of this, the legislative position did not arise as a main topic of discussion in engagement with the public and staff members. However, in seeking to promote cross-centre working in population data science, particularly across jurisdictional boundaries where the law may differ, or in working with sharing identifiable data, it will be essential to ensure compliance with all relevant legislative and regulatory requirements. This should include a review of the relevant instruments in discussion with legal experts and data controllers. This is also important because data centres will have differing operating models and means of data access which must be taken into account to determine the optimum combined model for an instance of cross-centre working to ensure safety and maximum data utility [23, 31].

### Recommendations

From the work we have carried out, we propose the following recommendations for advancing cross-centre working between data centres.

1. There is a need for dedicated resources to curate and make data available for reuse across data centres. This should be embodied more clearly in funder policies and funding specifications.
2. Since many of the practical issues encountered in cross-centre working are held in common, we propose an international forum to share experiences and solutions. This could be facilitated by a network such as the IPDLN.
3. Specific context-based data provider and public engagement is required to take into account varying operating models and proposed changes, as well as local culture and variations in the political landscape.
4. The particulars of legislative, regulatory and data provider due diligence approval processes call for dialogue inclusivity with all relevant parties within and across the particular scenarios under consideration.
5. Academic institutions and funding bodies need to place quantifiable value on the reuse of data. This could be enacted by means of a trackable dataset asset number.
6. The work of all staff contributing significantly to cross-centre working needs to be acknowledged. As well as proper inclusion on articles and funding bids, this will need to accommodate the most relevant performance indicators by staff role.
7. Cultural issues can be notoriously difficult to address in general. We advocate strong leadership to champion the cause. To be successful this needs to be demonstrated by the tangible commitment of funding bodies and policy makers.
8. Because of the risk of becoming bogged down, we advise not trying to overcome all the perceived barriers before moving forward, but to use a combination of solutions by balancing necessary barrier removal with incentivisation.

### Limitations

We acknowledge the following limitations in this study. Our public engagement was with one group and considered one cross-centre working scenario. We did not specifically engage with data providers due to limited project resources. The main interactions with data centre staff were with those employed by the Farr Institute in the UK. As such they might not be representative of all staff at the Farr centres, or more widely. With such a large scope of possibilities, it was not possible to consider all types of organisations that provide access to data, nor all types of dataset. We also limited the focus to anonymised data; a consideration of the issues for cross-centre working using data not classified as anonymous (in all its permutations) would warrant a separate study. However, the findings could inform further work with a range of stakeholders.

## Conclusion

Cross-centre working in population data science has the potential to open up new opportunities for research if the challenges can be successfully overcome. The ACoRN study has provided new information by exploring the barriers and solutions to cross-centre working. The views of a public group were that, providing the data are anonymised and safeguarded, cross-centre working involving data being moved between established centres is considered acceptable. Data centre staff provided valuable insights on the need to address dedicated resourcing, practical issues, information governance and culture. The challenges in achieving meaningful cross-centre working and it becoming the norm are multi-faceted, complex and inter-related. Through collaboration, it should be possible to avoid duplication of effort and work towards common frameworks, whilst allowing for jurisdictional and centre-specific variations. Our final conclusion is that achieving success will require on-going commitment, engagement and an academic culture change. Only time will tell whether cross-centre working in population data science will be a widespread norm or a nicety.

## References

[1] McGrail KM, Jones KH, Akbari A, Bennett T, Boyd A, Carinci F et al (2018) A Position Statement on Population Data Science: The science of data about people, IJPDS, 3:1, 10.23889/ijpds.v3i1.415 PMC8142960 34095517

[2] Institute of Clinical and Evaluative Sciences (2019) https://www.ices.on.ca/Publications

[3] Secure Anonymous Information Linkage Databank (2019) https://saildatabank.com/saildata/sail-publications/

[4] Population Health Research Network (2019) https://www.phrn.org.au/publications/research-publications/

[5] Medical Research Council (2019) Data sharing policy https://mrc.ukri.org/documents/pdf/mrc-data-sharing-policy/

[6] Economic and Social Research Council (2019) Research data policy https://esrc.ukri.org/files/about-us/policies-and-standards/esrc-research-data-policy/

[7] Canadian Institutes of Health Research (2018) Strategy for Patient-Oriented Research, National Data Platform http://www.cihr-irsc.gc.ca/e/50891.html

[8] Health Data Research UK (2019) https://www.hdruk.ac.uk/about/

[9] Farr Institute of Health Informatics Research (2018) http://farrinstitute.org/

[10] European Union (2016) General Data Protection Regulation https://gdpr-info.eu/

[11] HM Government (2018) UK Data Protection Act http://www.legislation.gov.uk/ukpga/2018/12/notes/division/6/index.htm

[12] HM Government (1998) UK Data Protection Act https://www.legislation.gov.uk/ukpga/1998/29/contents

[13] Jones KH, McNerney CL and Ford DV (2014) Involving consumers in the work of a data linkage research unit. International Journal of Consumer Studies, 1 2014, 38:1:45-51, 10.1111/ijcs.12062

[14] Secure Anonymous Information Linkage Databank (2019) https://saildatabank.com/about-us/public-engagement/

[15] Farr Institute of Health Informatics Research (2017) http://farrinstitute.org/news/informatics-for-health-2017-international-delegates-meet-for-inter-disciplinary-conference

[16] Piwowar HA, Becich MJ, Bilofsky H and Crowley R.S (2008) Towards a Data Sharing Culture Recommendations for Leadership from Academic Health Centers, PLoS Med, 5(9): e183. 10.1371/journal.pmed.0050183 18767901 PMC2528049

[17] Van Panhuis WG, Paul P, Emerson C, Grefenstette J, Wilder R, Herbst A et al (2014) A systematic review of barriers to data sharing in public health, BMC Public Health, 14: 1144. 10.1186/1471-2458-14-1144 25377061 PMC4239377

[18] Pisani E and Abouzahr C (2010) Sharing Health Data: Good Intention Are Not Enough, Bull World Health Organ, 88(6): 462-6. 10.2471/BLT.10.078956 20539861 PMC2878150

[19] McGuirk PM, O’Neill PM and Mee KJ (2015) Effective Practices for Interagency Data Sharing: Insights from Collaborative Research in a Regional Intervention, AJPA, 74(2): 199-211. 10.1111/1467-8500.12098

[20] Smith R and Roberts I (2016) Time for sharing data to become routine: the seven excuses for not doing so are all invalid, F1000Res, Apr 29(5): 781. 10.12688/f1000research.8422.1 PMC4909097 27347380

[21] Federer LM, Lu Y-L, Joubert DJ, Welsh, J and Brandys B (2015) Biomedical Data Sharing and Reuse: Attitudes and Practices of Clinical and Scientific Research Staff, PloS One, 10(6): e0129506. 10.1371/journal.pone.0129506 26107811 PMC4481309

[22] International Journal of Population Data Science (2018) IJPDS Special issue: Cross-Centre Working https://ijpds.org/issue/view/10

[23] Song J, Elliot E, Morris AD, Kerssens JJ, Akbari A, Thompson S and Lyons R (2018) A case study in distributed team science in research using electronic health records. IJPDS Special issue: Cross-Centre Working, 3:3:3, 10.23889/ijpds.v3i3.442 PMC8142956 34095524

[24] Lugg-Widger FV, Angel L, Cannings-John R, Hood K Hughes K, Moody G and Robling R (2018) Challenges in accessing routinely collected data from multiple providers in the UK for primary studies: Managing the morass. IJPDS Special issue: Cross-Centre Working, 3:3:2, 10.23889/ijpds.v3i3.432 PMC8142952 34095522

[25] Katz A, Enns J, Wong ST, Williamson T, Singer A, McGrail KM, et al. Challenges Associated with Cross-Jurisdictional Analyses using Administrative Health Data and Primary Care Electronic Medical Records in Canada. IJPDS Special issue: Cross-Centre Working, 3:3:6, 10.23889/ijpds.v3i3.437 PMC8142948 34095523

[26] Lix L, Ayles J, Bartholomew S, Cooke C, Ellison J, Emond V et al (2018) The Canadian Chronic Disease Surveillance System: A model for collaborative surveillance. IJPDS Special issue: Cross-Centre Working, 3:3:5, 10.23889/ijpds.v3i3.433 PMC7299467 32935015

[27] Southern DA, James MT, Wilton SB, DeKoning L, Quan H, Kundtson ML and Ghali WA (2018) Expanding the impact of a longstanding Canadian cardiac registry through data linkage: challenges and opportunities. IJPDS Special issue: Cross-Centre Working, 3:3:9, 10.23889/ijpds.v3i3.441 PMC7299492 32935018

[28] Groome PA, McBride ML, Jiang L, Kendell C, Decker KM, Grunfeld E et al (2018) Lessons Learned: It Takes a Village to Understand Inter-Sectoral Care Using Administrative Data across Jurisdictions. IJPDS Special issue: Cross-Centre Working, 3:3:8, 10.23889/ijpds.v3i3.440 PMC7299469 32935017

[29] Butler AL, Smith M, Jones W, Adair CE, Vigod S, Kurdyak P and Lesage A (2018) Multi-province epidemiological research using linked administrative data: a case study from Canada. IJPDS Special issue: Cross-Centre Working, 3:3:4, 10.23889/ijpds.v3i3.440 PMC7299461 32935019

[30] Jones KH, Heys S, Tingay K, Jackson P and Dibben C (2019) The Good, the Bad, the Clunky: Improving the use of Administrative Data for Research. IJPDS 4:1:03, 10.23889/ijpds.v4i1.587 PMC7479922 32935024

[31] Jones KH and Ford DV (2018) Population Data Science: Advancing the safe use of population data for public benefit. Epidemiol Health. 2018; e2018061. 10.4178/epih.e2018061 PMC6367205 30703857

